# Microfluidic on-chip production of microgels using combined geometries

**DOI:** 10.1038/s41598-021-81214-7

**Published:** 2021-01-15

**Authors:** Hamed Shieh, Maryam Saadatmand, Mahnaz Eskandari, Dariush Bastani

**Affiliations:** 1 Department of Chemical and Petroleum Engineering, Sharif University of Technology, Tehran, Iran; 2 Department of Biomedical Engineering, Amirkabir University of Technology, Tehran, Iran

**Keywords:** Engineering, Drug delivery

## Abstract

Microfluidic on-chip production of microgels using external gelation can serve numerous applications that involve encapsulation of sensitive cargos. Nevertheless, on-chip production of microgels in microfluidic devices can be challenging due to problems induced by the rapid increase in precursor solution viscosity like clogging. Here, a novel design incorporating a step, which includes a sudden increase in cross-sectional area, before a flow-focusing nozzle was proposed for microfluidic droplet generators. Besides, a shielding oil phase was utilized to avoid the occurrence of emulsification and gelation stages simultaneously. The step which was located before the flow-focusing nozzle facilitated the full shielding of the dispersed phase due to 3-dimensional fluid flow in this geometry. The results showed that the microfluidic device was capable of generating highly monodispersed spherical droplets (CV < 2% for step and CV < 5% for flow-focusing nozzle) with an average diameter in the range of 90–190 μm, both in step and flow-focusing nozzle. Moreover, it was proved that the device could adequately create a shelter for the dispersed phase regardless of the droplet formation locus. The ability of this microfluidic device in the production of microgels was validated by creating alginate microgels (with an average diameter of ~ 100 μm) through an external gelation process with on-chip calcium chloride emulsion in mineral oil.

## Introduction

The microfluidic technique is a promising technology that has attracted growing interest in recent years. The ability to provide controlled environmental conditions, continuous flow system and laminar flow at the microscale length have contributed to the growth of microfluidics technology for many multidisciplinary applications such as nanotechnology, biotechnology, biochemistry, physics, and engineering^1,2^.

In recent years, the generation of emulsions' particles and microparticles using microfluidic platforms has gained considerable attention due to their significant advantages over conventional bulk methods^3^. In the conventional methods, such as emulsion polymerization and precipitation polymerization, there is no or very little precise control over particles' size distribution, crosslink density uniformity, and morphology, especially for producing complicated geometries such as onion layers or multiple emulsions^4^. However, the droplet-based microfluidics technique enables pragmatic control in the fabrication of emulsions and microparticles. Monodispersed microdroplets in microfluidics can provide a compartment in which species or reactions can be isolated from the surrounding environment, so it is suitable for quantitative studies and offers a significant number of opportunities in chemical and biological research and applications^5^. Also, droplet-based microfluidics technology affords the production of monodispersed and shape-controlled microgels, which has various applications in tissue engineering and cell biology, drug delivery, and separation processes^2,6^.

Microfluidic generation of the microgels typically consists of two stages: microfluidic emulsification of precursor solutions and gelation of the generated droplets. The latter stage can be done either on-chip or off-chip mode. The on-chip gelation process is generally preferred since it has several advantages over off-chip gelation, including simplification of the manipulation of the microgels' morphologies, paving the way for loading a wide variety of cargos, and continuous generation of the microgels with a high degree of monodispersity^7^. However, some challenges have remained in the on-chip gelation process. A rapid increase of the viscosity in the precursor solutions during gelation is one of the possible difficulties in this method. As a result, the microchannel and nozzles may be clogged. Also, in these systems, to control the dispersity and morphology of generated microgels, there should be a time delay between the emulsification and gelation processes^7^. To accomplish this task, several research groups have come up with some novel ideas. Wang et al. used a water stream in the central microchannel to prevent the mixing of ionic triblock copolymers with a different charge, which flows in the side microchannels before flow-focusing nozzle^8^. Similar to this work, Mazutis et al. prevented premature gelation of alginate by using a water stream. This stream of water flowed between the precursor solution and crosslinking solution, which contained calcium chloride as crosslinker^9^. Another method for postponing the gelation process is reducing the pH of the crosslinker solution to slow down gelation kinetics^10^. Marquis et al. developed a system in which droplet gelation of pectin mixed with calcium carbonate was induced by the diffusion of acetic acid from the oil phase to the droplets where the resulting pH decrease inside the droplets led to calcium bridging and biopolymer gelation^11^. Then Utech et al., in similar but more reliable work, took advantage of a water-soluble complex of EDTA and calcium to avoid premature gelation. Calcium, which was in a complex form, cannot crosslink the alginate, but when acid is added to the solution, the dissociation of the complex was triggered by pH reduction, which results in the gelation of the droplets^12^. Headen et al. and Weaver et al. employed two streams of a shielding oil phase to protect the macromer solution from contact with the crosslinker-laden oil phase. Since crosslinker could not reach the macromere before flow instability occurred, monodisperse, spherical droplets were formed. After droplet formation, the crosslinker diffused into droplets and cross-linked the PEG-4MAL macromer into the hydrogel network^13,14^.

Regarding the first stage in the microfluidic generation of the microgels, which is the microfluidic emulsification of precursor solutions, a wide variety of geometries and configurations have been proposed and studied previously. The most common geometries in droplet formation devices include cross-flow (T-junction), flow focusing, and co-flow junctions^1,4^. Furthermore, other geometries like step emulsification are used for the microfluidic droplet generation platforms, which are capable of producing highly monodispersed droplets compared to other geometries^4,15^. Few previous studies proposed combined configurations of these geometries in order to benefit the advantages of each geometry. Priest et al. implemented a step after a T-junction in their microfluidic device to broaden the range of applicability of the device for droplet formation^16^. Chan et al. developed a device that utilized a combination of flow focusing and step emulsification geometries. In their device, there was a step after a flow-focusing nozzle to make sure of the dispersed phase breakup^17^. Again, Liu et al. made use of a step after a flow-focusing nozzle in their microfluidic device for external gelation of alginate droplets^18^. However, in almost all of these researches, either those utilizing a combined configuration or those using a single geometry, the desired droplet generation process (production of spherical droplets in a dripping mode) has been confined to a limited range of flow rates^19^.

In this study, we developed a microfluidic device for the production of highly monodispersed spherical microgels. In the proposed design, challenges of both stages in the microfluidic generation of microgels, namely emulsification and gelation stages, were addressed. Concerning the first stage, we utilized a combination of step emulsification and flow-focusing geometries. In the developed design, a step was implemented before the flow-focusing nozzle, and both of them were used for generating droplets. It was shown that locating a step before the flow-focusing nozzle improved the shielding of the dispersed phase and extended the range of flow rates for droplet formation.

Regarding the second stage, we used a sheltered phase, co-flowing the dispersed phase before a step for separating emulsification and gelation stages in time. This setup allowed on-chip external gelation of precursor droplets without facing the complications of on-chip gelation in microfluidic droplet generators. Here, the external gelation method was used due to its advantages over internal gelation in the production of microgels especially for encapsulation purposes^20,21^. The 3-dimensional fluid flow in step geometry helps complete and 3-dimensional shielding of the dispersed phase, which in turn facilitates external gelation of precursor solution droplets and on-chip production of microgels. To the best of our knowledge, such a design for the on-chip generation of microgels has not been reported before. Here we synthesized microgels with alginate as a model polymer, which its external gelation for the synthesis of microgels in microfluidic devices has not been studied much before^18^.

## Materials and methods

### Materials

Light mineral oil was purchased from Sigma Aldrich. SPAN 80 (sorbitan monooleate) was obtained from Tokyo Chemical Industry (Japan). Calcium chloride and alginate were purchased from Sigma Aldrich. Deionized water (DI water) was obtained from a laboratory purification system. Polydimethylsiloxane (PDMS) (Sylgard 184) was purchased from Dow Corning Corporation.

### Device fabrication

**Figure 1:**
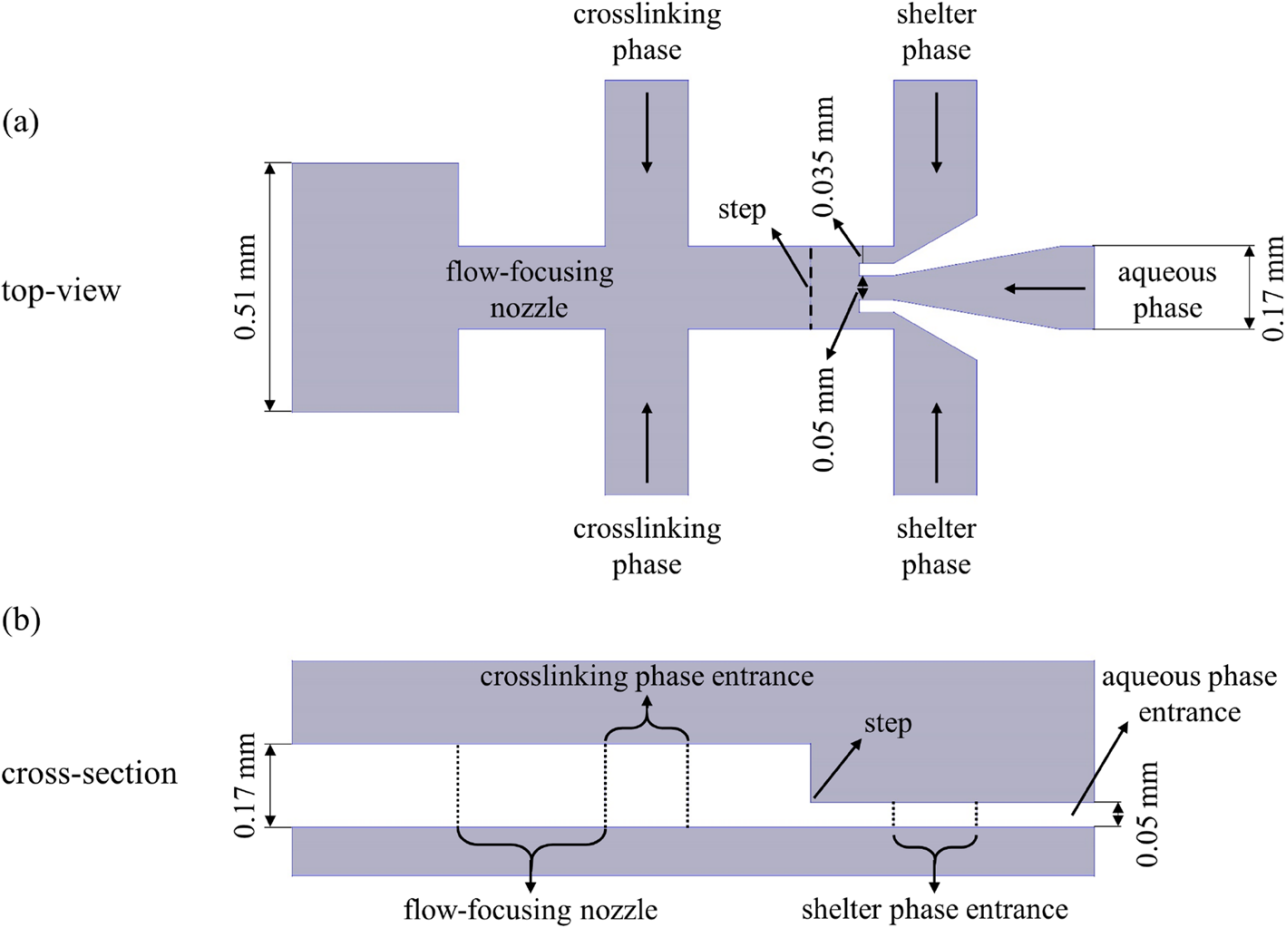
Schematic illustration of the designed microfluidic system. (**a**) top-view. (**b**) cross-section.

The silicone mold was made by MMT-co (Tehran, Iran) using the SU-8 photoresist and standard photolithography method. The microfluidic device was fabricated using standard soft lithography method^22^. In this way, PDMS and curing agent were mixed with a 10:1 ratio, and the mixture was degassed in a vacuum desiccator using a vacuum pump (Platinum Series Vacuum Pumps, JB). The obtained mixture was poured onto the mold and degassed again. The mold containing the PDMS was placed in an oven at 60 °C for seven hours to bake the PDMS. The PDMS was detached from the mold after the baking process, and the inlets and outlet were punched using a 1.5 mm skin punch (Ribble International Ltd.).In order to seal the microchannels, the PDMS slab was bonded to a glass slide (25 mm × 75 mm) using the plasma treatment method (air pressure: 300 mTorr, time: 15 s, and power: 100 W). Finally, for better bonding between PDMS and glass and enhancing the hydrophobicity of the PDMS, the device was placed in an oven at 60 °C for two hours^12,23^. The prepared PDMS device has three inlets for a dispersed aqueous phase containing alginate and two continuous phases containing mineral oil (Fig. 1). The aqueous phase and shelter phase channels had a height of 50 μm, and their width was 50 and 35 μm, respectively. The height of the step and flow-focusing nozzle was 120 and 170 μm, respectively, and their width was the same and equal to 170 μm.

### Droplet formation

For performing the droplet formation experiments, three phases (aqueous phase, shelter phase, and crosslinking phase) were injected through the device channels by using two syringe pumps (SP1000HSM, Fnm co., Iran). The syringe pumps were connected to the device with silicone tubes having an internal diameter of 0.6 mm. In these experiments, both the shelter phase and the crosslinking phase had the same composition containing light mineral oil with 2% SPAN 80 as the surfactant. Moreover, DI water was used as an aqueous phase. The range of flow rates for the aqueous phase (Q_a_), shelter phase (Q_s_), and crosslinking phase (Q_c_) was 1–48 μL min^−1^, 0.44–108 μL min^−1^, and 6–60 μL min^−1^, respectively. In the proposed design (Fig. 1a), when the aqueous phase moves toward the flow-focusing nozzle, the shelter phase, which moves in the same direction, wraps the aqueous phase in the shielding section (the section before the flow-focusing nozzle) until the droplets of the aqueous phase form in the step (the dashed line in Fig. 1a) or the flow-focusing nozzle. If the aqueous droplets do not form in the step, the middle flowing fluid reaches the flow-focusing nozzle, where the crosslinking phase cuts this fluid and the aqueous phase droplets form. After that, the droplets move through the outlet channel and then leave the device via the outlet.

### Generation of alginate microgels

In order to generate alginate microgels, the aqueous phase contained 1% of alginate in DI water, and the crosslinking phase comprised of an emulsion (1:10 v/v) of calcium chloride aqueous solution as a crosslinking agent in light mineral oil with 2% of SPAN 80. The concentration of calcium chloride in the emulsion was in the range of 4–10 mol L^−1^. For preparing the crosslinking phase, the emulsion of calcium chloride in mineral oil was stirred overnight. This preparation method led to the formation of a highly uniformed emulsion, which was very stable and did not become separated in the syringe during the experiments. After running the device and forming aqueous droplets, the calcium, which presents in the crosslinking phase, penetrates these droplets and starts to crosslink the alginate in these droplets, leading to the formation of alginate microgels.

It should be noted that the shelter phase wraps the aqueous phase before the formation of aqueous droplets in the step or the flow-focusing nozzle. Therefore, the crosslinker could not reach the aqueous phase before uniformed aqueous droplets form and retain their shape to a large extent. The generated microgels after leaving the device were collected in microtubes using a silicone tube. In order to separate the collected microgels, they were washed with a one molar calcium chloride solution and then centrifuged (Hettich, Germany) for 1 min at a speed of 500 rpm.

### Image analysis of droplets

The droplet formation process was visualized using an inverted microscope (TCM 400, LABOMED, USA). In order to investigate the monodispersity of generated droplets, the coefficient of variation (CV) of 30 droplets was evaluated in each experiment. The ImageJ analysis software was used to analyze the captured images and evaluate the CV, droplets diameter, and microgels diameter.

## Results and discussion

### Design of microfluidic device

As mentioned, the objective of this study was to produce monodispersed microgels using a sheltered phase. In order to reach this objective, the angle of contact between the aqueous phase and the shelter phase was set to zero. The idea behind this design was that a standard method for generating droplets is to set a 90° angle between two immiscible phases^24^. As a result, in this work, the contact angle between immiscible phases (aqueous phase and shelter phase) was set to its lowest possible value (zero angles) in order to minimize the exerting shear force from the shelter phase to the aqueous phase and prevent the generation of droplets in the shielding section. Finally, this zero angle caused the proper shielding of the aqueous phase (will be shown in “Shielding of aqueous phase” section) and the prevention of undesirable formation of aqueous droplets. Moreover, there is a step in this design between the shielding section and flow-focusing nozzle, enabling the fabricated device to generate droplets in a different range of flow rates. Thus, the device can generate droplets using two different geometries (at the step and flow-focusing nozzle), each operating in a different range of flow rates.

### Generating monodispersed droplets using microfluidic device

**Figure 2:**
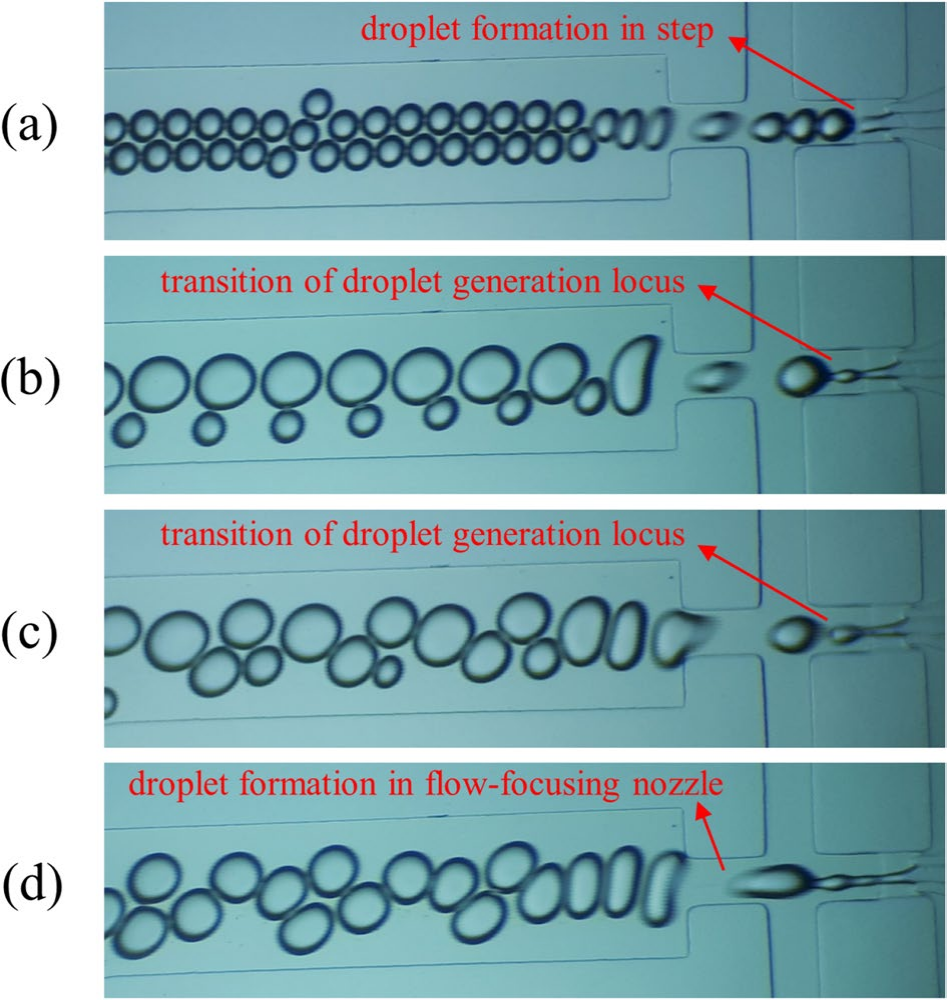
Transition of droplet generation locus (from step (**a**) to flow focusing nozzle (**d**)) with increasing Q_a_ and Q_s_ at a constant ratio (α = 1) and a specific value of Q_c_ (Q_c_ = 30 μL/min). (**a**) Q_a_ = 18 μL/min. (**b**) Q_a_ = 24 μL/min. (c) Q_a_ = 30 μL/min. (d) Q_a_ = 36 μL/min.

**Figure 3:**
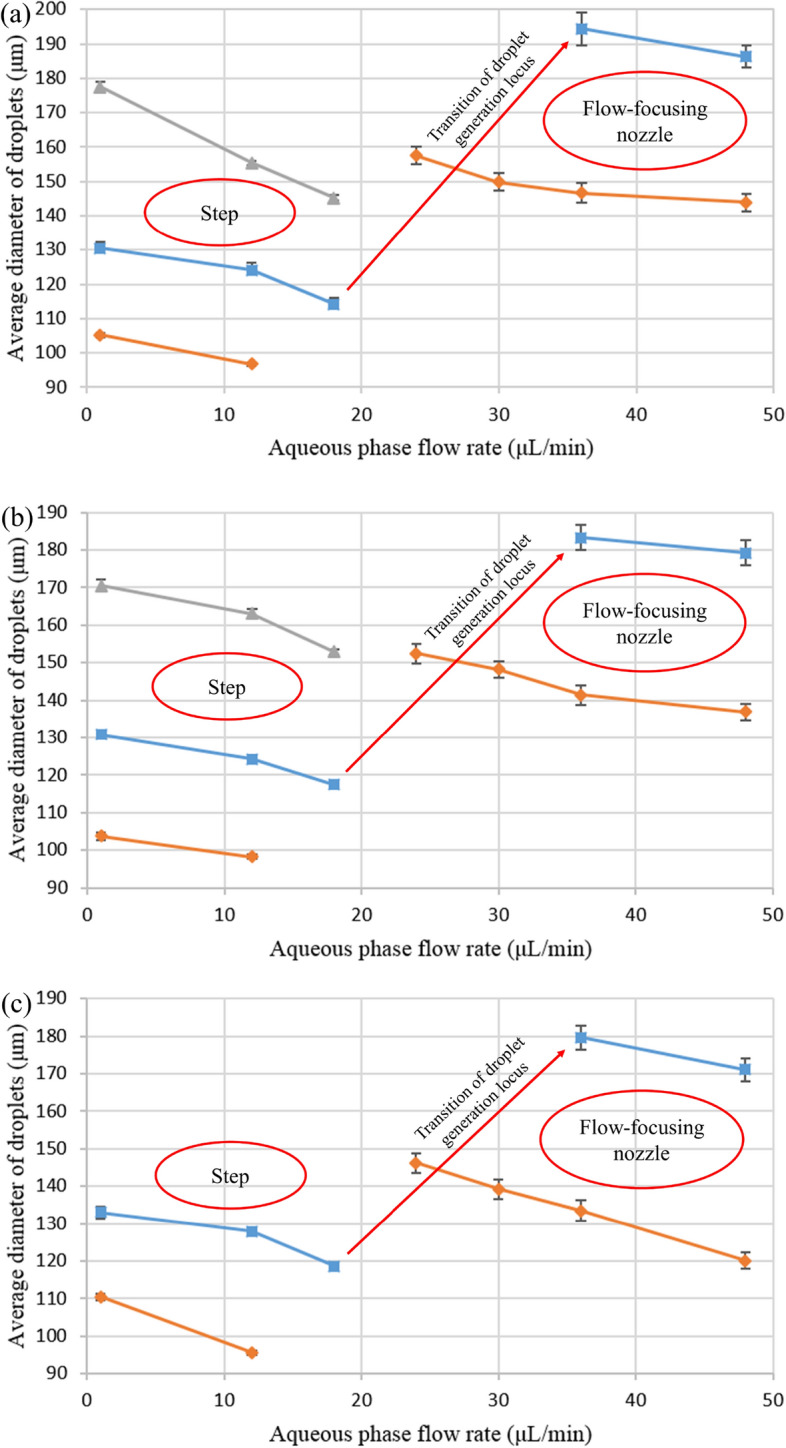
Average diameter of generated droplets versus flow rate of aqueous phase. α: ■ = 1, ♦ = 2.25, and ▲ = 0.44. (**a**) Q_c_ = 6 μL/min. (**b**) Q_c_ = 30 μL/min. (**c**) Q_c_ = 60 μL/min. Error bars indicate standard deviation for average diameter of droplets (n = 2).

As it was cleared in our design, the developed geometry had a step between the shielding section and the flow-focusing nozzle. Based on droplet generation tests, it turned out that the design is capable of generating droplets both in the flow-focusing nozzle and in the step (Fig. 2a,d) while yielding the desired shielding of the aqueous phase. In previous studies in which the microfluidic device had a combined configuration, including a step, it was shown that the locus of droplet generation is dependent on flow rates of phases entering the device before the step and their ratio^16,17^. Therefore, it was expected that, in this work, the locus of the droplet generation should be dependent on Q_a_, Q_s_, and their ratio (α = Q_s_/Q_a_). The first phenomenon which was expected and observed here was that droplet generation occurred in step at small values of Q_a_ and Q_s_. Then by increasing Q_a_ and Q_s_ (at constant α), the locus of droplet generation moved to the flow-focusing nozzle [Figs. 2 and 3 (shown by red arrows)]. In this section, different sets of flow rates vary in either α or Q_c_, or both of them.

Another observation during droplet generation experiments was that by increasing α, the transition of droplet generation locus from step to flow-focusing nozzle occurred in lower flow rates, and vice versa (Fig. 3). Moreover, by comparing Fig. 3a–c, it can be concluded that Q_c_ had no impact on the transition of droplet generation locus from the step to the flow-focusing nozzle. For instance, in all of those three figures (having different Q_c_ values), the droplet generation locus moved from step to flow-focusing nozzle after increasing Q_a_ from 12 μL/min to 24 μL/min at α = 2.25. Similarly, for other sets of flow rates, it can be observed that the movement of the droplet generation locus is independent of Q_c_.

Therefore, there are two compelling factors in determining the locus of droplet formation. The impact of the first factor, which is the value of Q_a_ and Q_s_, is noticeable^16,17^. In fact, at constant α, by increasing Q_a_, the aqueous phase velocity increases. Hence, the aqueous phase will not have enough time to split up in step, which leads to the reduction of droplet formation tendency in step. As a result, the aqueous phase moves toward a flow-focusing nozzle, and it will cut off there by the crosslinking phase. For example, in Fig. 3a, by increasing Q_a_ from 18 μL/min to 36 μL/min at α = 1, the droplet generation locus moved from step to flow-focusing nozzle. The same trend can be observed for other values of α and Q_c_. Another reason for this observation is that more shear stress exerts from the shelter phase to the aqueous phase by increasing Q_a_ and Q_s_, which drives the aqueous phase toward the flow-focusing nozzle.

Regarding the effect of the second factor, which is the value of α, it can be inferred that this factor determines the locus of droplet formation by altering the amount of exerted shear stress between the aqueous and shelter phase. It means that, at constant Q_a_, the larger the value of α is, the more shear stress is exerted from the shelter phase to the aqueous phase, which prevents the aqueous phase split up in step. For instance, at Q_c_ = 6 μL/min (Fig. 3a) when α increased from 1 to 2.25 start point of droplet generation locus transition decreased from Q_a_ = 18 μL/min to Q_a_ = 12 μL/min. Due to this fact, the aqueous phase tendency to pass step increases by increasing α. Hence, the transition of droplet generation locus from step to flow-focusing nozzle occurs in lower flow rates.

Another observation in these tests was that the transition of droplet generation locus from step to flow-focusing nozzle occurs in a range of flow rates rather than in a specific flow rate. As shown in Fig. 3, data for some average Q_a_ values have not been reported (shown by red arrows). Such as in Fig. 3a, for α = 1, there is no data corresponding to Q_a_ values between 18 μL/min to 36 μL/min. These flow rates depict the ranges in which the transition of droplet generation locus from step to flow-focusing nozzle occurred. The aqueous phase droplets formed during this transition were polydispersed with a high coefficient of variation (CV > 10%, Fig. 2b,c); therefore, their data has not been reported in Fig. 3. Indeed, the data points before these unreported ranges are related to aqueous droplets formed in step, and the data after them are depicting aqueous droplets formed in the flow-focusing nozzle.

Furthermore, as can be observed from the error bars depicted in Fig. 3, for a specific set of flow rates, the droplets which were produced in step had a lower CV compared to those which were generated in the flow-focusing nozzle. However, all of the droplets, whether they were produced in step or the flow-focusing nozzle, had an ideal monodispersity (CV < 5%). The low CV of generated droplets indicates the high capability of the proposed microfluidic design for the synthesis of highly monodispersed droplets. Moreover, compared to the flow-focusing nozzle, the diameter of generated droplets in step was smaller for a particular set of flow rates (the range for each droplet generation locus is indicated with a red circle in Fig. 3). Also, it should be noted that for a specific set of flow rates, the diameter of droplets decreased by increasing Q_a_ (hence increasing α) regardless of the locus of droplet synthesis. For instance, at Q_c_ = 6 μL/min (Fig. 3a) and α = 1, the diameter of droplets decreased from 130 μm to 115 μm when Q_a_ increased from 3 to 18 μL/min (in the step region). The same trend was observed for the flow-focusing nozzle region where droplets diameters decreased from 195 μm to 185 μm when Q_a_ increased from 36 to 48 μL/min. The same trend is observable at other values of Q_c_ and α. These results were compatible with research works in which two co-flowing phases flow before droplet generation locus^13,25^. The reason could probably be the more shear force, which was exerted to the aqueous phase from the shelter phase due to its faster velocity in higher flow rates. This increased force could reduce the time that the aqueous phase spent in droplet formation loci and resulted in the formation of smaller droplets. This observation contrasts with those researches in which just a dispersed phase flows before droplet generation locus^26–28^.

Moreover, by increasing Q_c_, for example, from Q_c_ = 6 μL/min (Fig. 3a) to Q_c_ = 30 μL/min (Fig. 3b) at α = 1 and Q_a_ = 3 μL/min, the average diameter of droplets did not change significantly (~ 130 μm). However, when Q_c_ increased from 6 to 30 μL/min at α = 1 and Q_a_ = 36 μL/min, the average diameter of droplets decreased from 195 to 185 μm. This observation suggests that Q_c_ had no significant impact on the diameter of droplets that were produced in the step. However, the value of Q_c_ could influence the diameter of droplets, which were generated in the flow-focusing nozzle, and by increasing Q_c_, the diameter of generated droplets decreased as it could be expected^27,29,30^.

Also, at α = 0.44, data in Fig. 3a,b has been reported for a limited range of Q_a_, and average diameters in Fig. 3c have not been mentioned for the whole range of Q_a_. Indeed, that data has not been reported because either no droplets were produced or aqueous phase slugs were generated in those sets of flow rates.

### Shielding of aqueous phase

**Figure 4:**
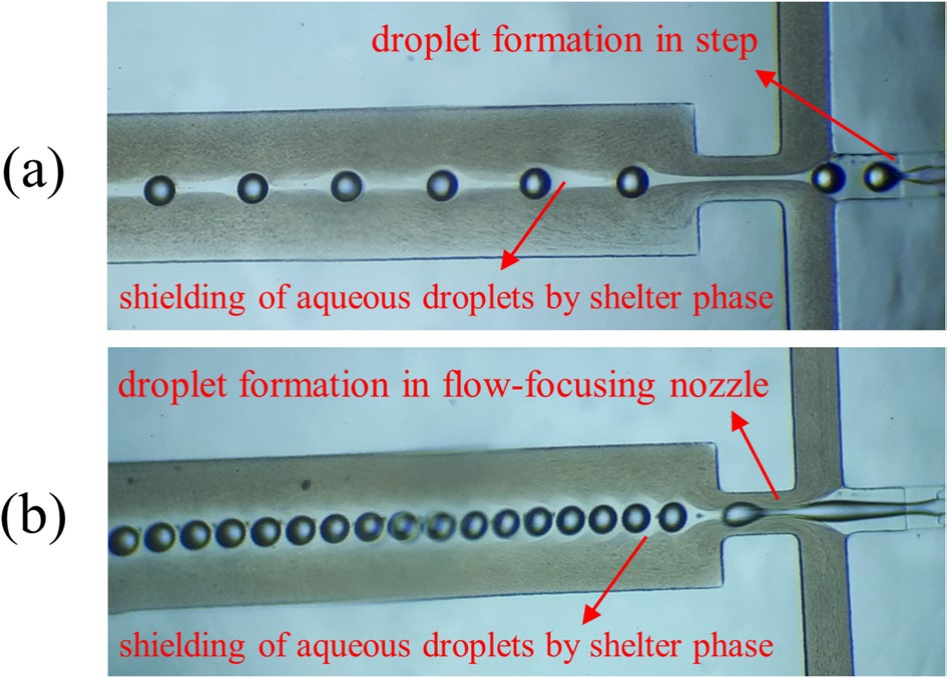
Shielding of aqueous phase droplets by shelter phase when aqueous droplets were formed in (**a**) step and (**b**) flow-focusing nozzle.

A unique characteristic of microfluidics is that it allows achieving any desirable configurations of co-flowing phases, which is almost impossible to attain using macro systems. During the experiments performed in this study, it was observed that the developed microfluidic chip is capable of providing a good shield for the aqueous phase with the shelter phase. This result can be approved quickly by inspecting the images, which were obtained during microgel production tests. As can be observed in Fig. 4, regardless of the formation of aqueous phase droplets in either step (Fig. 4a) or flow-focusing nozzle (Fig. 4b), the shelter phase created an observable shielding between the aqueous phase droplets and crosslinking phase. In previous studies in which a kind of shielding was utilized, the dispersed phase droplets formed only in one locus or using one geometry^11,13,14,31^. However, in the present study, there were two droplet generation loci. The notable point is that the shielding created by the shelter phase was independent of the droplet generation locus, which demonstrated that the developed chip was adequately designed.

### Alginate microgels synthesis

**Figure 5:**
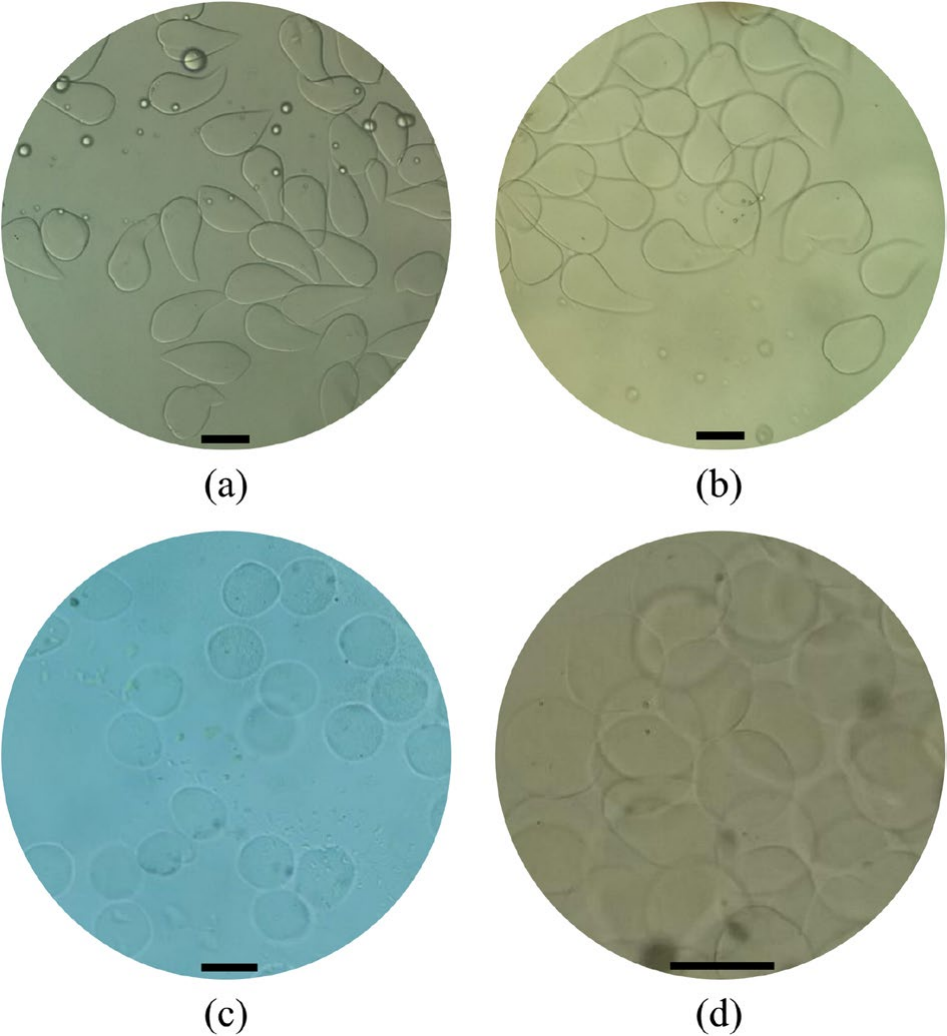
Alginate microgels were synthesized by using the designed microfluidic device. Calcium chloride concentration in crosslinking phase emulsion was (**a**) 4 mol/L, (**b**) 6 mol/L, (**c**) 8 mol/L, and (**d**) 10 mol/L. Scale bars are 100 μm.

**Figure 6:**
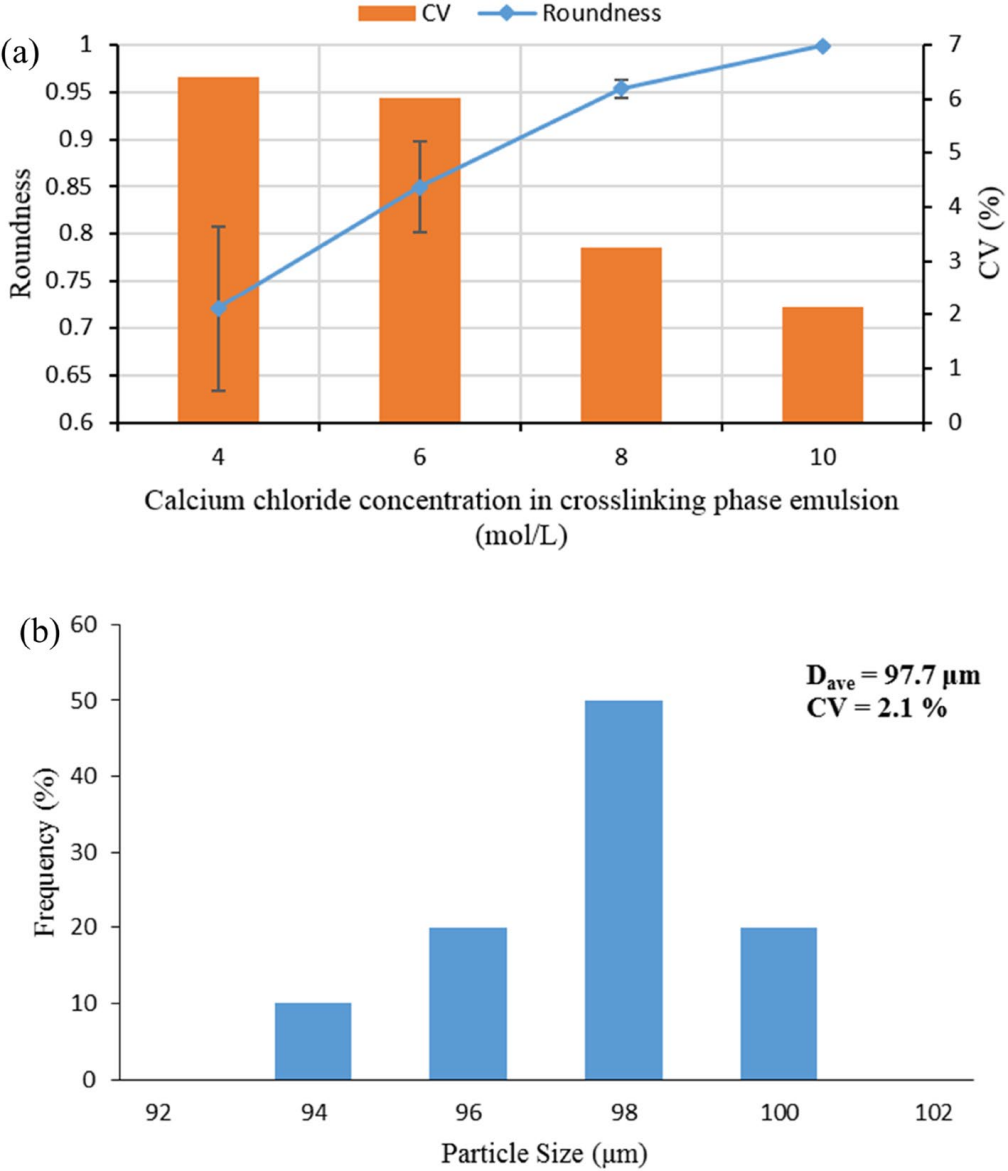
(**a**) Effect of calcium chloride concentration on roundness and CV of alginate microgels. (**b**) Size distribution of alginate microgels produced with a calcium chloride concentration of 10 mol/L.

In order to produce alginate microgels, as it was stated in Sect. 2.4, the crosslinking phase contained an emulsion (1:10 v/v) of calcium chloride aqueous solution as a crosslinking agent in light mineral oil with 2% of SPAN 80 as the surfactant. The production process of microgels in both the step and the flow-focusing nozzle is depicted in Fig. 4. In these experiments, the effect of calcium chloride concentration in the crosslinking phase emulsion on the capability of synthesis of microgels was investigated. In order to do this, the concentration of calcium chloride varied between 4 and 10 mol/L. In low concentrations of calcium chloride, non-spherical microgels with a drop-like shape were produced (Figs. 5a,b, 6a). Production of drop-like (also called the teardrop or tail-shaped) microgels indicates that all of the gelation processes or most of them were occurred outside of the microfluidic device^32,33^. An observation that confirms this theory is the formation of spherical alginate droplets during these experiments. As shown in Fig. 4, the produced alginate droplets were spherical, and if their gelation process occurred entirely in the microfluidic device, the generated microgels should be spherical. Even if the gelation process does not occur entirely inside the microfluidic device and just a shell forms around the droplets (gelation process occurs partly), provided that this shell has an adequate thickness, it can maintain the spherical shape of microgels after leaving the microfluidic device^34^.

As depicted in Figs. 5 and 6a, the shape of generated microgels became more spherical with a lower CV by increasing the concentration of calcium chloride compared to those with low calcium concentration (Fig. 5c,d). This observation shows that at higher concentrations of calcium chloride, a larger portion of the gelation process occurs inside the microfluidic device, and microgel production becomes a more on-chip process. This observation is probably due to the faster diffusion of more calcium ions from small droplets of aqueous solution in the crosslinking phase emulsion into the alginate droplets at higher concentrations of calcium chloride. However, this will cause faster gelation of alginate droplets, leading to on-chip gelation of these droplets and maintaining their spherical shape after gelation. Finally, with increasing calcium chloride concentration, up to 10 mol/L highly monodispersed microgels with a spherical shape were produced (Figs. 5d, 6). Moreover, the production of spherical microgels depicts a potential and significant advantage of the combined-configuration used in this study over other geometries used in literature, which is independence on complex and sometimes difficult pretreatments of microchannels (e.g. for manipulating the hydrophobicity of microchannels) during or after fabrication process of the device^34^. Furthermore, these results demonstrate greater reliability of the proposed method for the production of spherical microgels than other studies existing in literature, which suffered from the production of drop-like particles even after implementing several steps in the production process^21^.

## Conclusion

On-chip production of spherical microgels through an external gelation process was achieved by using a designed microfluidic device. A novel design that incorporates a step before a flow-focusing nozzle was used for droplet formation. Utilizing a combination of two geometries extended the range of flow rates for droplet formation. Moreover, a sheltered phase was used for dispersed phase shielding, which allowed separation of emulsification and gelation in time. Placing the step before the flow-focusing nozzle facilitated the complete shielding of the dispersed phase, making it possible to perform the external gelation process in the best way. The effect of flow rates of different phases on the droplet formation process (diameter of droplets and locus of droplet formation) was investigated. The device's ability to generate highly monodispersed spherical microgels was verified by producing alginate (as a model polymer) microgels through external gelation of alginate droplets with calcium chloride emulsion in mineral oil. Furthermore, the influence of calcium chloride concentration in the emulsion on the gelation process was explored. Overall, the results of experiments showed the ability of advanced design in the generation of highly monodispersed spherical microgels. The proposed design is exceptionally suitable for microfluidic encapsulation of sensitive cargos using microgels due to the used shielding phenomenon and on-chip external gelation process.

## References

[1] Damiati S, Kompella UB, Damiati SA, Kodzius R (2018). Microfluidic devices for drug delivery systems and drug screening. Genes.

[2] Chung BG, Lee K-H, Khademhosseini A, Lee S-H (2012). Microfluidic fabrication of microengineered hydrogels and their application in tissue engineering. Lab Chip.

[3] Liu AL, García AJ (2016). Methods for generating hydrogel particles for protein delivery. Ann. Biomed. Eng..

[4] Li W (2018). Microfluidic fabrication of microparticles for biomedical applications. Chem. Soc. Rev..

[5] Theberge AB (2010). Microdroplets in microfluidics: An evolving platform for discoveries in chemistry and biology. Angew. Chem. Int. Ed..

[6] Dendukuri D, Doyle PS (2009). The synthesis and assembly of polymeric microparticles using microfluidics. Adv. Mater..

[7] Tumarkin E, Kumacheva E (2009). Microfluidic generation of microgels from synthetic and natural polymers. Chem. Soc. Rev..

[8] Wang CX (2016). Non-covalent microgel particles containing functional payloads: Coacervation of PEG-based triblocks via microfluidics. ACS Appl. Mater. Interfaces.

[9] Mazutis L, Vasiliauskas R, Weitz DA (2015). Microfluidic production of alginate hydrogel particles for antibody encapsulation and release. Macromol. Biosci..

[10] Foster GA (2017). Protease-degradable microgels for protein delivery for vascularization. Biomaterials.

[11] Marquis M, Davy J, Cathala B, Fang A, Renard D (2015). Microfluidics assisted generation of innovative polysaccharide hydrogel microparticles. Carbohydr. Polym..

[12] Utech S (2015). Microfluidic generation of monodisperse, structurally homogeneous alginate microgels for cell encapsulation and 3D cell culture. Adv. Healthc. Mater..

[13] Headen DM, Aubry G, Lu H, García AJ (2014). Microfluidic-based generation of size-controlled, biofunctionalized synthetic polymer microgels for cell encapsulation. Adv. Mater..

[14] Weaver JD (2019). Synthetic poly (ethylene glycol)-based microfluidic islet encapsulation reduces graft volume for delivery to highly vascularized and retrievable transplant site. Am. J. Transpl..

[15] Huang X (2017). Collective generation of milliemulsions by step-emulsification. RSC Adv..

[16] Priest C, Herminghaus S, Seemann R (2006). Generation of monodisperse gel emulsions in a microfluidic device. Appl. Phys. Lett..

[17] Chan EM, Alivisatos AP, Mathies RA (2005). High-temperature microfluidic synthesis of CdSe nanocrystals in nanoliter droplets. J. Am. Chem. Soc..

[18] Liu Y, Tottori N, Nisisako T (2019). Microfluidic synthesis of highly spherical calcium alginate hydrogels based on external gelation using an emulsion reactant. Sens. Actuators B Chem..

[19] Azimi-Boulali J, Madadelahi M, Madou MJ, Martinez-Chapa SO (2020). Droplet and particle generation on centrifugal microfluidic platforms: A review. Micromachines.

[20] Chan LW, Lee HY, Heng PW (2006). Mechanisms of external and internal gelation and their impact on the functions of alginate as a coat and delivery system. Carbohydr. Polym..

[21] Yang C-H (2020). Facile synthesis of highly tunable monodispersed calcium hydroxide composite particles by using a two-step ion exchange reaction. RSC Adv..

[22] Xia Y, Whitesides GM (1998). Soft lithography. Annu. Rev. Mater. Sci..

[23] Jeong H-H, Jin SH, Lee BJ, Kim T, Lee C-S (2015). Microfluidic static droplet array for analyzing microbial communication on a population gradient. Lab Chip.

[24] Wang JT, Wang J, Han JJ (2011). Fabrication of advanced particles and particle-based materials assisted by droplet-based microfluidics. Small.

[25] Agarwal P (2013). One-step microfluidic generation of pre-hatching embryo-like core–shell microcapsules for miniaturized 3D culture of pluripotent stem cells. Lab Chip.

[26] Kim C, Park KS, Kim J, Jeong SG, Lee CS (2017). Microfluidic synthesis of monodisperse pectin hydrogel microspheres based on in situ gelation and settling collection. J. Chem. Technol. Biotechnol..

[27] Huang K-S, Lai T-H, Lin Y-C (2006). Manipulating the generation of Ca-alginate microspheres using microfluidic channels as a carrier of gold nanoparticles. Lab Chip.

[28] Um E, Lee D-S, Pyo H-B, Park J-K (2008). Continuous generation of hydrogel beads and encapsulation of biological materials using a microfluidic droplet-merging channel. Microfluid. Nanofluid..

[29] Siltanen C (2016). Microfluidic fabrication of bioactive microgels for rapid formation and enhanced differentiation of stem cell spheroids. Acta Biomater..

[30] Hsu MN (2018). Smart hydrogel microfluidics for single-cell multiplexed secretomic analysis with high sensitivity. Small.

[31] Madrigal JL (2018). Microgels produced using microfluidic on-chip polymer blending for controlled released of VEGF encoding lentivectors. Acta Biomater..

[32] Kim C (2009). Rapid exchange of oil-phase in microencapsulation chip to enhance cell viability. Lab Chip.

[33] Lin YS, Yang CH, Hsu YY, Hsieh CL (2013). Microfluidic synthesis of tail-shaped alginate microparticles using slow sedimentation. Electrophoresis.

[34] Samandari M, Alipanah F, Javanmard SH, Sanati-Nezhad A (2019). One-step wettability patterning of PDMS microchannels for generation of monodisperse alginate microbeads by in Situ external gelation in double emulsion microdroplets. Sens. Actuators B Chem..

